# Exercise versus usual care after non-reconstructive breast cancer surgery (UK PROSPER): multicentre randomised controlled trial and economic evaluation

**DOI:** 10.1136/bmj-2021-066542

**Published:** 2021-11-11

**Authors:** Julie Bruce, Bruno Mazuquin, Alastair Canaway, Anower Hossain, Esther Williamson, Pankaj Mistry, Ranjit Lall, Stavros Petrou, Sarah E Lamb, Sophie Rees, Emma Padfield, Raghavan Vidya, Alastair M Thompson, Prevention of Shoulder Problems Trial (PROSPER) Study Group, C Lait, C Hegarty, M van Laar, C Harkin, L Chowdhury, H Richmond, L Betteley, C Srikesavan, M Newman, J Moser, A Tomlins, K McEvoy, P G Roy, R Soulsby, F Hoar, JS McNicholas, I Azmy, S Mitchell, C Osborne, J Donnelly, E Babu, N van de Ploeg, T Sircar, K Makam, E McLoughlin, M Noblet, S Soumian, A Thorne, L Wagstaff, L Fort, C Rushton, M Evans, A Simpson, L Ridgeway, D Pilgrim, L Graves, K Jones, J Holt, N Ekers, A McMullen, C Lindsay, S Wright, R Bower, S Horne, S Rook, N Redfern, A Allen, S Greenwood, E McLoughlin, A Stephenson, R Conway, C Edley, J Pitts, N Layte, H Thomas, C Johnson, A Heath, L Beadle, R Scarisbrick, D DeBeer, D Dungey, Y Stokes, S Calloway, H Brown, N Clarke, C Daffern, A Willis, H Adjei, C Muthiah

**Affiliations:** 1 Warwick Clinical Trials Unit, Division of Health Sciences, University of Warwick, Coventry, UK; 2 Faculty of Health, Psychology and Social Care, Manchester Metropolitan University, Manchester, UK; 3 Institute of Statistical Research and Training (ISRT), University of Dhaka, Dhaka, Bangladesh; 4 Nuffield Department of Orthopaedics Rheumatology and Musculoskeletal Sciences, University of Oxford, Oxford, UK; 5 Nuffield Department of Primary Care Health Sciences, University of Oxford, Oxford, UK; 6 Institute of Health Research, University of Exeter, Exeter, UK; 7 Royal Wolverhampton NHS Trust, Wolverhampton, UK; 8 Department of Surgery, Dan L Duncan Comprehensive Cancer Center, Baylor College of Medicine, Houston, TX 77030, USA

## Abstract

**Objective:**

To evaluate whether a structured exercise programme improved functional and health related quality of life outcomes compared with usual care for women at high risk of upper limb disability after breast cancer surgery.

**Design:**

Multicentre, pragmatic, superiority, randomised controlled trial with economic evaluation.

**Setting:**

17 UK National Health Service cancer centres.

**Participants:**

392 women undergoing breast cancer surgery, at risk of postoperative upper limb morbidity, randomised (1:1) to usual care with structured exercise (n=196) or usual care alone (n=196).

**Interventions:**

Usual care (information leaflets) only or usual care plus a physiotherapy led exercise programme, incorporating stretching, strengthening, physical activity, and behavioural change techniques to support adherence to exercise, introduced at 7-10 days postoperatively, with two further appointments at one and three months.

**Main outcome measures:**

Disability of Arm, Hand and Shoulder (DASH) questionnaire at 12 months, analysed by intention to treat. Secondary outcomes included DASH subscales, pain, complications, health related quality of life, and resource use, from a health and personal social services perspective.

**Results:**

Between 26 January 2016 and 31 July 2017, 951 patients were screened and 392 (mean age 58.1 years) were randomly allocated, with 382 (97%) eligible for intention to treat analysis. 181 (95%) of 191 participants allocated to exercise attended at least one appointment. Upper limb function improved after exercise compared with usual care (mean DASH 16.3 (SD 17.6) for exercise (n=132); 23.7 (22.9) usual care (n=138); adjusted mean difference 7.81, 95% confidence interval 3.17 to 12.44; P=0.001). Secondary outcomes favoured exercise over usual care, with lower pain intensity at 12 months (adjusted mean difference on numerical rating scale −0.68, −1.23 to −0.12; P=0.02) and fewer arm disability symptoms at 12 months (adjusted mean difference on Functional Assessment of Cancer Therapy-Breast+4 (FACT-B+4) −2.02, −3.11 to −0.93; P=0.001). No increase in complications, lymphoedema, or adverse events was noted in participants allocated to exercise. Exercise accrued lower costs per patient (on average −£387 (€457; $533) (95% confidence interval −£2491 to £1718; 2015 pricing) and was cost effective compared with usual care.

**Conclusions:**

The PROSPER exercise programme was clinically effective and cost effective and reduced upper limb disability one year after breast cancer treatment in patients at risk of treatment related postoperative complications.

**Trial registration:**

ISRCTN Registry ISRCTN35358984.

## Introduction

Breast cancer treatments can affect the lymphatic and musculoskeletal systems of the torso and upper limb. Adverse sequelae after surgery and radiotherapy targeting the axilla are common, and up to one third of women experience restricted range of motion in the shoulder, chronic pain, and lymphoedema, limiting quality of life and delaying recovery.^1^ ^2^ In the UK, guidelines for non-reconstructive breast surgery advocate gradual reintroduction of upper limb mobility, and referral to physiotherapy is recommended if problems develop.^3^ However, the optimal timing, intensity, safety, and impact of postoperative exercise are uncertain, particularly in women undergoing axillary clearance surgery or axillary/supraclavicular radiotherapy, who are at increased risk of developing shoulder and upper limb related disability. Concerns include that early or overly vigorous exercise may increase risks of postoperative wound complications and lymphoedema.^4^ ^5^ Systematic reviews highlight the paucity of evidence for the introduction of postoperative range of motion and strengthening exercises on functional outcomes.^4^ ^6^ ^7^ Many published studies excluded higher risk groups, the very population that may benefit most from targeted support to prevent postoperative upper limb disability. No rigorous randomised controlled trials of sufficient sample size have been conducted to show the safety or clinical effectiveness of early exercise after breast cancer surgery among patients at the highest risk of developing upper limb disability. Few trials have examined function, health related quality of life, and other patient reported outcomes over the longer term. A systematic review found that evidence on the cost effectiveness of exercise and physiotherapy interventions for breast cancer patients was sparse.^8^ Before this study, no published (or registered) multicentre trial had evaluated whether early, structured, progressive postoperative exercise is clinically effective and cost effective for patients at higher risk of shoulder problems after targeted treatment to the axilla, supraclavicular area, or both (surgery/radiotherapy).

The aim of the UK Prevention of Shoulder Problems Trial (PROSPER) was to investigate the effects of an exercise programme compared with best practice usual care for women at high risk of upper limb disability after treatment for breast cancer. Outcomes included upper limb function, complications (pain, wound related complications, lymphoedema), health related quality of life, and cost effectiveness.

## Methods

### Study design

The UK PROSPER trial was a pragmatic, superiority, multicentre, randomised controlled trial undertaken at 17 National Health Service (NHS) cancer centres. The trial protocol (version 2.1, 2017), a detailed description of the development of the intervention, and an embedded qualitative study have been published.^9^ ^10^ ^11^ A protocol amendment was approved in 2018 to allow qualitative interviews with physiotherapists delivering the exercise intervention.

### Participants

Women aged 18 years or older with newly diagnosed, histologically confirmed invasive or non-invasive breast cancer who were scheduled for surgery and considered to be at high risk of upper limb disability after surgery were eligible. We defined women as being at high risk if they were scheduled to undergo planned axillary node clearance or to have planned radiotherapy to the axilla or supraclavicular fossa, had a high body mass index (≥30), had existing shoulder problems as per PROSPER criteria (supplementary box S1), had any subsequent axillary surgery after sentinel lymph node biopsy, or had planned axillary or supraclavicular radiotherapy within six weeks of primary surgery. Patients informed of the need for axilla/supraclavicular radiotherapy were permitted postoperative entry to the trial only if the exercise intervention could be started within six weeks of the primary surgery.

### Randomisation and masking

We randomly allocated participants (1:1) to usual care only or usual care plus structured exercise, using a computer generated sequence to ensure allocation concealment, via a secure, centralised telephone randomisation service administered by an independent programmer. The sequence was prepared by programmers and tested by the trial statistician. We used three stratification variables: recruitment centre, first or repeat surgery, and whether the patient was informed of the need for radiotherapy within six weeks of surgery. The nature of the exercise intervention meant that we did not blind participants or physiotherapists. Senior research team members were blind to treatment allocation for the duration of the trial. A statistician independent of the core trial team did the final statistical analyses.

### Interventions

Participants randomised to best practice usual care were provided with written information leaflets recommending postoperative exercises and generic postoperative advice freely available from the UK charity Breast Cancer Care.^12^ ^13^ Women allocated to usual care received no further intervention other than these leaflets, which were provided during preoperative clinics. Women randomised to the exercise programme also received these leaflets and were then referred to physiotherapy for a supervised, structured exercise programme. The programme was based on accepted principles of exercise prescription and progression; it was underpinned by behavioural change strategies, including motivational interview techniques, to encourage participants to adhere to exercise. The aim of the intervention was to restore range of movement in the shoulder, improve strength, and increase physical activity. We developed the intervention from literature review and consultation with stakeholders, including breast cancer patients undergoing active treatment, community cancer support groups, physiotherapists, rehabilitation specialists, and surgeons. The research team developed a draft intervention and refined it at an intervention development meeting with stakeholders. We produced a menu of upper limb exercises targeting shoulder flexion, abduction, and abduction with external rotation, from which the treating physiotherapist could prescribe an individually tailored programme.^9^ We produced materials to support adherence to exercise and integration of behaviour change techniques (for example, exercise booklet and exercise diary). We piloted the exercise programme with patients with newly diagnosed breast cancer.

The final exercise programme was fully manualised with documented pathways for clinical assessment and exercise prescription, with guidance for management of postoperative complications. It consisted of at least three face-to-face therapy sessions with a trained physiotherapist (seven to 10 days, one month, and three months postoperatively), with participants permitted a maximum of six sessions over one year. The additional sessions could be delivered in person or by telephone.

The first session at seven to 10 days postoperatively was a one hour assessment by the physiotherapist. An individually tailored daily range of movement exercises was prescribed targeting shoulder flexion, abduction, and abduction with external rotation. Behavioural support strategies were used (collaborative goal setting, assessing confidence to exercise, exercise diary, identifying barriers, and facilitators to exercise). These were reviewed at subsequent sessions.

Follow-up sessions were of 30 minutes duration. From one month postoperatively, shoulder strength was assessed and strengthening exercises were prescribed to target deficits in shoulder flexion, abduction, and external rotation strength. Participants were given resistance bands (Therabands) for home use; these were used to tailor the level of resistance for each participant. Strength exercises were carried out at least twice a week. Participants were asked to gradually increase their physical activity with the aim of undertaking 150 minutes of moderate intensity activity per week, in line with American Cancer Society guidance.^14^ At review sessions, the programme was progressed by increasing sets, repetitions, and resistance load of exercises and by progressing duration and intensity of physical activity. A detailed description of the exercise programme has been published separately.^10^

### Procedures

After preoperative screening by oncology teams to identify women at higher risk of developing shoulder problems, eligible patients were provided with written trial information. Invitation packs included a baseline questionnaire, consent form, and usual care information leaflets. A trained member of the research team obtained informed consent. Participants randomly allocated to exercise were referred for the first physiotherapy assessment within seven to 10 days of surgery. In this pragmatic trial, any other rehabilitation input beyond usual care was left to the discretion of the oncology team, as per normal clinical practice, and captured in questionnaires. We collected primary and secondary patient reported outcomes and resource use by using postal questionnaires at baseline (pre-randomisation) and six weeks, six months, and 12 months post-randomisation. Follow-up questionnaires were mailed from and returned to the Warwick Clinical Trials Unit, independently of oncology teams. All baseline measures were ascertained preoperatively, except for late entry participants identified as being at high risk recruited within six weeks of surgery (recorded pre-randomisation).

### Exercise intervention training and fidelity

Trial research staff trained 44 physiotherapists, with at least two physiotherapists trained from each participating NHS hospital. Physiotherapists were trained in trial procedures and the content and delivery of the exercise programme, including behavioural support strategies and motivational interviewing techniques. For each participant randomised to the exercise programme, physiotherapists completed treatment logs to record appointments, strength assessments, prescribed exercises, and overall progress with the programme. Research physiotherapists undertook intervention quality assurance checks by observing at least one therapy session with each physiotherapist, with consent from the trial participant. Verbal and written feedback on adherence to the study protocol was provided to all physiotherapists carrying out the exercise intervention.

### Outcomes

The primary outcome was upper limb function assessed using the Disabilities of the Arm, Shoulder and Hand (DASH) questionnaire at 12 months post-randomisation.^15^ Breast cancer treatments targeting the axilla and shoulder can affect upper limb function generally, leading to difficulty with activities such as writing, dressing, opening and closing jars, and lifting shopping bags. DASH is 30 item patient reported scale that ranges from no disability (score 0) to most severe disability (100).^15^ It includes 21 items of function, six items for symptoms, and three items on social/role function. DASH is recommended for measurement of upper extremity disorders in breast cancer survivors.^16^ ^17^

Secondary outcomes, captured in follow-up questionnaires over 12 months, were DASH subscales (activity limitations, impairment, and participation restriction)^18^; postoperative pain (acute, chronic, and neuropathic pain),^9^ measured using a numerical rating scale, douleur neuropathique^19^; the Functional Assessment of Cancer Therapy-Breast+4 (FACT-B+4) arm symptom subscale^20^; complications (wound related, including surgical site infection, seroma, and wound healing); lymphoedema (Lymphoedema and Breast Cancer Questionnaire)^21^; and health related quality of life (measured by SF-12 and EQ-5D-5L).^22^ ^23^ We collected health and personal social service resource use data through self-report within postal questionnaires and extracted hospital resource use data from NHS Digital hospital episode statistics. Research staff extracted surgical and treatment related data from medical records after 12 months’ follow-up and transferred them to the Warwick Clinical Trials Unit via secure NHS data transfer pathways.

### Sample size

The minimum clinically important difference for adults with acute or chronic upper extremity orthopaedic or rheumatological conditions for the DASH questionnaire is five to 10 points, suggesting moderate improvement.^24^ ^25^ In a breast cancer population, a small Dutch trial found a mean group difference of seven points at six months after a three month exercise intervention.^26^ We selected a seven point difference in DASH scores at 12 months to account for the preventive approach rather than treatment of an established, chronic condition and to allow for the pragmatic trial design, whereby some participants in the control arm may be exposed, by serendipity, to other active interventions. At 80% power and 5% type 1 error rate on a two sided test, we needed to randomise 242 participants. The sample was inflated for therapist effects, with an estimated nine participants per therapist, yielding an intracluster coefficient of 0.01 and design effect of 1.05, giving 256 participants. We allowed 25% loss to account for loss to follow-up over one year, giving a sample size of 350 participants.

### Statistical and health economic analysis

The primary statistical analysis was an intention to treat analysis that included all participants in their randomised groups. We used an ordinary linear regression model to compare the primary outcome of DASH score at 12 months between treatment groups. We did a complier average causal effect analysis for the primary outcome for randomised participants who were fully adherent to the exercise programme, defined a priori as having at least three physiotherapy contacts. We analysed change in DASH score from baseline to six and 12 months by treatment arm and plotted mean changes (with 95% confidence intervals) graphically. Models were adjusted for age, baseline DASH score, breast surgery, axillary surgery, radiotherapy, and chemotherapy. We used similar methods to analyse the SF-12 data, comparing scores between treatment arms. We used multiple imputation to examine the effect of missing DASH data. We calculated “strength and work capacity” to reflect dose of strengthening exercises prescribed, defined as the product of repetitions and sets prescribed at each session; these data will be reported in a separate publication.

Within trial economic evaluation estimated the cost effectiveness of the exercise programme compared with usual care after breast cancer surgery (see economic evaluation in supplementary materials). The primary health economic analysis took the form of a cost-utility analysis, expressed in terms of incremental cost per quality adjusted life year (QALY) gained and incremental net monetary benefit. We captured intervention costs by using case report forms, and physiotherapists and the trial team collected intervention delivery data. We measured broader resource use with an adapted client service receipt inventory at six and 12 months’ follow-up. We calculated costs by combining resource use data with unit costs from standard sources such as the Personal Social Services Research Unit cost compendia.^27^ The primary measure of health consequence in the economic evaluation was the QALY. We used the Van Hout algorithm to derive utility values from the EQ-5D-5L, measured at baseline, six months, and 12 months.^23^ We estimated QALYs by using linear interpolation between utility values with the trapezoid rule. Additionally, for a subsample of 242 participants (those with 12 months of complete data post-randomisation before the NHS Digital data cut-off date of 31 March 2018), we sourced secondary care use data on inpatient hospital spells and outpatient attendances over the duration of the trial from hospital episode statistics for financial years 2015-18 for sensitivity analyses.

The health economic analysis used the intention to treat principle. In line with guidance from the National Institute for Health and Care Excellence (NICE),^28^ the analysis adopted an NHS and Personal Social Services perspective. The price year for the analysis was 2015, which was when the trial intervention materials were developed. The health economic analysis used a 12 month time horizon with no discounting of costs or outcomes. We used multiple imputation to correct for missing data, assumed to be missing at random. To maximise the use of available data, we used imputation at the component level (for example, for each healthcare cost variable and EQ-5D-5L assessment at each time point). We imputed costs and EQ-5D-5L utility scores jointly using chained equations and predictive mean matching; the imputation model included age, ethnicity, marital status, employment status, and recruiting site as covariates. We used hierarchical linear models to analyse the single cost and QALY endpoints and a hierarchical net benefit regression framework to jointly examine costs and consequences. We characterised uncertainty around cost effectiveness by using net benefit plots and cost effectiveness acceptability curves, in addition to multiple sensitivity analyses (see supplementary materials). We estimated the probability of cost effectiveness of the exercise programme for NICE thresholds of £20 000 and £30 000 per QALY gained. We provide further details on the methods for the economic evaluation and sensitivity analyses in supplementary materials.

### Study monitoring

Trial steering and data monitoring committees reviewed safety, quality, and masked data at six monthly intervals and approved the statistical analysis plan and protocol. We did no interim analyses, but the trial steering committee/data monitoring committee could halt the trial for safety or ethical concerns. We obtained appropriate permissions and paid any required fees for use of copyright protected materials.

### Patient and public involvement

Patients were involved at multiple stages, providing input to the design, management, and conduct of the trial. Patients and members of the public, from community cancer support groups, co-produced and reviewed exercise intervention materials.^10^ We included a lay member on the trial steering committee.

## Results

**Fig 1:**
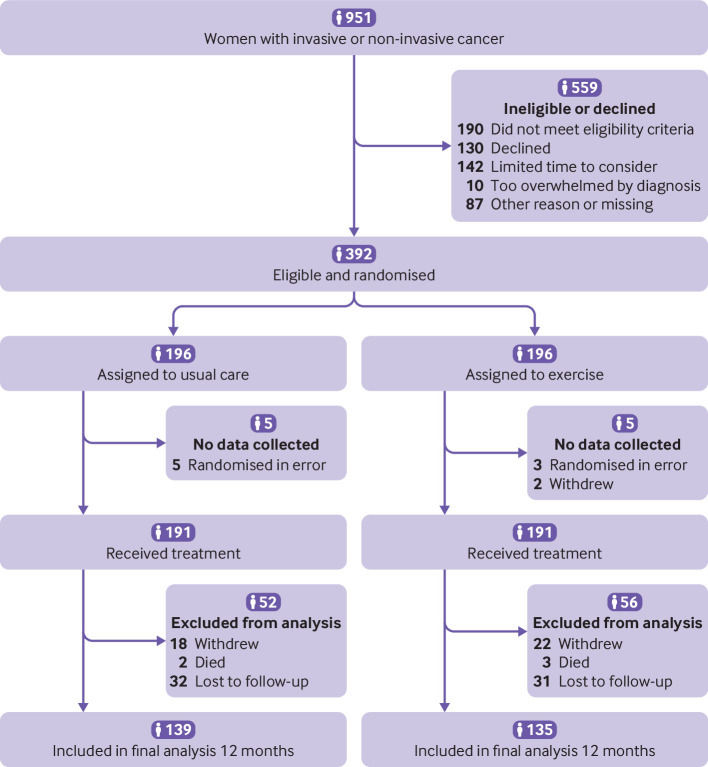
Flowchart of participants. No data were collected for 10 participants: eight randomised in error with bilateral or breast reconstruction surgery and two immediate withdrawals due to change of mind

Between 26 January 2016 and 31 July 2017, of 951 women screened, 392 (41%) were randomly allocated to exercise and usual care (n=196) or usual care alone (n=196). Ten (3%) patients were randomised in error and excluded from analyses as no data were collected: eight exclusions were due to surgical exclusions (bilateral/immediate breast reconstruction surgery), and two women withdrew immediately at the point of randomisation (fig 1). The remaining 382 participants were allocated to either usual care (n=191; 50%) or the exercise programme (n=191; 50%). The mean age of recruited participants was 58.1 (SD 12.1; range 28-88) years. Overall, most participants had axillary node clearance (327/382; 86%) and/or axillary/supraclavicular radiotherapy (317/382; 83%), and most (277/382; 73%) were overweight or obese at recruitment. One fifth (83/392; 21%) had a history of shoulder problems at recruitment. Table 1 shows participants’ characteristics by treatment group. Baseline patient reported outcome data were available for 350/382 (92%) of those allocated to treatment (175 per treatment group); 8% did not return baseline questionnaires.

**Table 1:** Participants’ treatment* and baseline characteristics. Values are numbers (percentages) unless stated otherwise

Characteristics	Usual care	Exercise	Total
**Randomised to treatment**	(n=191)	(n=191)	(n=382)
Mean (SD) age, years	57.8 (12.0)	58.4 (12.2)	58.1 (12.1)
Mean (SD) body mass index; (missing)	30.6 (7.2); (5)	29.9 (6.9); (6)	30.2 (7.0); (11)
Body mass index:			
<25	44 (23)	50 (26)	94 (25)
25-<30	51 (27)	53 (28)	104 (27)
≥30	91 (48)	82 (43)	173 (45)
Missing	5 (3)	6 (3)	11 (3)
Axillary surgery:			
Axillary node clearance	162 (85)	165 (86)	327 (86)
Sentinel lymph node biopsy	26 (14)	26 (14)	52 (14)
None	2 (1)	0	2 (<1)
Missing	1 (<1)	0	1 (<1)
Radiotherapy:			
Yes	166 (87)	151 (79)	317 (83)
No	14 (7)	26 (14)	40 (10)
Missing	11 (6)	14 (7)	25 (7)
Site of radiotherapy†:			
Breast	114 (60)	94 (49)	208 (54)
Chest wall	50 (26)	57 (30)	107 (28)
Axilla/supraclavicular area	62 (32)	51 (27)	113 (30)
Radiotherapy boost given	60 (31)	44 (23)	104 (27)
Chemotherapy given	118 (62)	108 (57)	226 (59)
Axillary node clearance and axillary/supraclavicular radiotherapy	65 (34)	53 (28)	118 (31)
**Baseline characteristics**‡	(n=175)	(n=175)	(n=350)
Marital status:			
Single	18 (10)	15 (9)	33 (9)
Married/cohabiting	127 (73)	124 (71)	251 (72)
Divorced/separated/widowed	30 (17)	34 (19)	64 (18)
Missing	0	2 (1)	2 (<1)
Education:			
School only	54 (31)	58 (33)	112 (32)
Work qualification	36 (21)	35 (20)	71 (20)
College or university	84 (48)	80 (46)	164 (47)
Missing	1 (<1)	2 (1)	3 (1)
Employment status:			
Full or part time employed	65 (37)	70 (40)	135 (39)
Self-employed	6 (3)	10 (6)	16 (5)
Retired	67 (38)	65 (37)	132 (38)
Housewife, mother/carer	16 (9)	6 (3)	22 (6)
Illness/disability/other	16 (9)	23 (13)	39 (11)
Missing	5 (3)	1 (<1)	6 (2)
Ethnicity:			
White	159 (91)	162 (93)	321 (92)
Asian	12 (7)	5 (3)	17 (5)
Afro-Caribbean	1 (<1)	2 (1)	3 (1)
Mixed	0 (0)	2 (1)	2 (<1)
Other	2 (1)	3 (2)	5 (1)
Missing	1 (<1)	1 (<1)	2 (<1)
Comorbidities:			
None	47 (27)	47 (27)	94 (27)
1-2	86 (49)	90 (51)	176 (50)
≥3	42 (24)	38 (22)	80 (23)
Any shoulder problem:			
Yes	29 (17)	45 (26)	74 (21)
No	120 (69)	105 (60)	225 (64)
Missing	26 (15)	25 (14)	51 (15)
Upper limb function:			
Mean (SD) DASH; (missing)	18.2 (19.8); (4)	19.5 (21.2); (8)	18.8 (20.5); (12)
Median (IQR) DASH	11.7 (1.7-30.0)	12.5 (2.5-30.8)	12.3 (1.7-30.2)
Neuropathic pain, DN4:			
No pain	95 (54)	89 (51)	184 (53)
*≤*3 (non-neuropathic pain)	57 (33)	63 (36)	120 (34)
>3 (neuropathic pain)	17 (10)	16 (9)	33 (9)
Missing	6 (3)	7 (4)	13 (4)
Mean (SD) pain intensity, NRS; (missing)	1.9 (2.5); (6)	1.9 (2.4); (13)	1.9 (2.4); (13)
Mean (SD) FACT-B+4; (missing)	2.7 (4.0); (0)	3.1 (4.2); (1)	2.9 (4.1); (1)
Lymphoedema, LBCQ:			
Arm feels heavy	38 (22)	43 (25)	81 (23)
Arm looks swollen	27 (15)	25 (14)	52 (15)
Arm heavy and swollen	20 (11)	17 (10)	37 (11)
Arm neither heavy not swollen	152 (87)	148 (85)	300 (86)
Missing	3 (2)	10 (6)	13 (4)
Health related quality of life:			
Mean (SD) EQ-5D-5L; (missing)	0.67 (0.22); (18)	0.68 (0.20); (16)	0.67 (0.2); (34)
Mean (SD) SF-12 PCS; (missing)	47.6 (11.6); (8)	46.8 (11.6); (7)	47.2 (11.6); (15)
Mean (SD) SF-12 MCS; (missing)	44.7 (11.7); (8)	46.8 (10.6); (7)	45.8 (11.2); (15)
Outside walking, days per week:			
Never or seldom (1-2)	38 (22)	46 (26)	84 (24)
Sometimes (3-4)	51 (29)	54 (31)	105 (30)
Often (5-7)	85 (49)	75 (43)	160 (46)
Missing	1 (<1)	0	1 (<1)
Strenuous sport/recreation, days per week:			
Never	134 (77)	132 (75)	266 (76)
Seldom (1-2)	26 (15)	25 (14)	51 (15)
Sometimes/often (≥3)	13 (7)	15 (9)	28 (8)
Missing	2 (1)	3 (2)	5 (1)
Mean (SD) confidence scores; (missing):			
Return to usual activities	7.5 (2.5); (2)	8.1 (2.3); (0)	7.8 (2.4); (2)
Return to physical activity	7.5 (2.3); (2)	8.0 (2.3); (0)	7.7 (2.3); (2)

DASH=Disability of Arm, Shoulder and Hand; DN4=Dolour Neuropathique-4; FACT-B+4=Functional Assessment of Cancer Therapy-Breast+4 arm scale; IQR=interquartile range; LBCQ=Lymphoedema and Breast Cancer Questionnaire; MCS=mental component summary score; NRS=numerical rating scale; PCS=physical component summary score.

* Treatment variables are most invasive surgery/adjuvant therapy by 12 month follow-up.

† Radiotherapy administered to >1 site; hence multiple response options possible.

‡ 350/382 (92%) completed baseline questionnaires pre-randomisation.

We obtained postal questionnaire data for 303/382 (79%) participants at six weeks, for 278/382 (73%) at six months, and for 274/382 (72%) at 12 months. Of those with complete baseline data, this equated to 303/350 (87%) at six weeks, 278/350 (79%) at six months, and 274/350 (78%) at 12 months. Uptake to the exercise programme was high, with 181/191 (95%) participants allocated to exercise engaging with the intervention by attending at least one physiotherapy appointment; 143/191 (75%) were fully adherent, attending three or more sessions. Physiotherapists had a total of 622 contacts (mean 3.7; median 3) with 181 participants who attended the exercise programme; 97% (603/622) of contacts were face to face, and the remainder were telephone reviews (19/622; 3%). From quality assurance checks, treatment was delivered according to the protocol; only one physiotherapist needed refresher training and support after training on the intervention. Strength exercises were prescribed from one month postoperatively. We observed an increase in mean dose of strength exercises for upper limb movement directions over time among participants adhering to exercise; these data will be reported separately.

**Fig 2:**
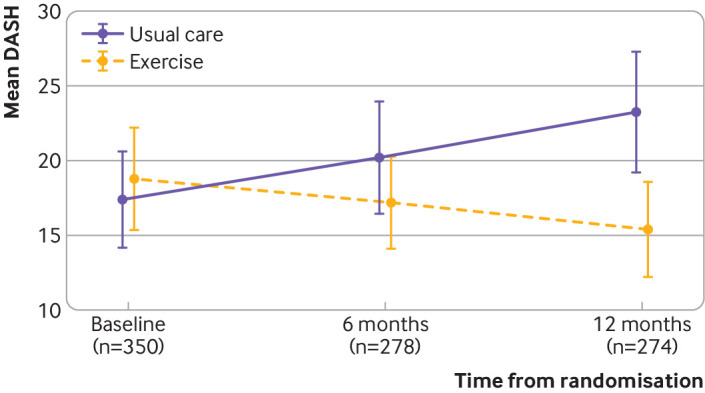
Mean (95% confidence interval) Disability of the Arm, Shoulder and Hand (DASH) scores by treatment group over time. DASH scores for intention to treat analysis, adjusted for age, baseline DASH, breast surgery, axillary surgery, radiotherapy, and chemotherapy. Higher scores indicate more disability

At 12 months post-randomisation, the mean DASH score was 16.3 (SD 17.6) for the exercise group and 23.7 (22.9) for the usual care group (table 2). The intention to treat analysis showed a statistically and clinically significant difference in mean DASH scores favouring the exercise intervention (unadjusted mean difference in DASH 7.34 (95% confidence interval 2.44 to 12.23; P<0.01); adjusted mean difference in DASH 7.81 (3.17 to 12.44; P=0.001) (table 2; fig 2). For the complier average causal effect analysis, in 143/191 (75%) participants who fully adhered to the intervention, we observed an increase in the between group difference in favour of the PROSPER exercise programme (adjusted mean difference in DASH 8.74 (3.77 to 13.71; P<0.001). We observed no differences in effect estimates after multiple imputation for data missingness.

**Table 2:** Disability of Arm, Shoulder, Hand (DASH) scores by treatment group

Time point, analysis	Usual care)			Exercise			Between group difference (95% CI)*			
	No	Mean (SD)		No	Mean (SD)		Unadjusted	P value	Adjusted	P value
6 months, ITT	125	20.8 (20.1)		134	18.0 (17.1)		2.76 (−1.79 to 7.31)	0.23	4.60 (0.30 to 8.90)	0.04
12 months, ITT (primary outcome)	138	23.7 (22.9)		132	16.3 (17.6)		7.34 (2.44 to 12.23)	<0.01	7.81 (3.17 to 12.44)	0.001
12 months, CACE		-			-		8.35 (2.85 to 13.84)	0.003	8.74 (3.77 to 13.71)	<0.001
Baseline to 6 months	118	−5.3 (19.4)		121	0.7 (17.8)		5.96 (1.23 to 10.70)	0.01	4.60 (0.31. 8.90)	0.04
Baseline to 12 months	130	−5.3 (19.7)		117	2.6 (19.7)		7.98 (3.03 to 12.92)	<0.01	7.81 (3.17 to 12.44)	0.001

Scores adjusted for age, baseline DASH, breast surgery, axillary surgery, radiotherapy, and chemotherapy. Mean differences in upper limb function favour exercise intervention.

CACE=complier average causal effect; ITT=intention to treat.

* Absolute mean difference between treatment groups.

We observed improvements at 12 months for all DASH subscales of activity limitations, participation restrictions, and impairment, favouring exercise compared with usual care (supplementary table S1). Postoperative pain intensity scores were lower at 12 months in participants randomised to exercise compared with usual care (adjusted mean difference in numerical rating scale −0.68, −1.23 to −0.12; P=0.02) (table 3). Acute postoperative pain scores at rest and on movement were lower in those randomised to exercise compared with usual care, but we observed no differences in mean pain intensity at six months (table 3). We observed fewer arm disability symptoms at both six months and 12 months, favouring exercise compared with usual care (adjusted mean difference in FACT-B+4 −2.02, −3.11 to −0.93; P=0.001) (table 3).

**Table 3:** Secondary outcomes of pain, arm symptoms, and lymphoedema by treatment group. Values are numbers (percentages) unless stated otherwise

Outcome	Usual care	Exercise	Estimate	P value
Mean (SD) pain intensity, NRS*:			Adjusted mean difference (95% CI)	
Pain at rest, 6 weeks	2.2 (2.5); (n=150)	1.6 (1.9) (n=153)	−0.58 (−1.09 to −0.07)	0.03
Pain on movement, 6 weeks	2.6 (2.6); (n=150)	2.1 (2.1) (n=153)	0.55 (−1.10 to −0.01)	0.04
Pain, 6 months	2.2 (2.3) (n=153)	2.0 (2.1) (n=148)	−0.17 (−0.70 to 0.35)	0.52
Pain, 12 months	2.6 (2.4) (n=139)	1.9 (2.0) (n=135)	−0.68 (−1.23 to −0.12)	0.02
			Adjusted odds ratio (95% CI)	
Moderate to severe, 6 weeks	46/150 (31)	28/153 (18)	1.90 (1.02 to 3.52)	0.04
Moderate to severe, 6 months	30/133 (23)	25/145 (17)	1.42 (0.72 to 2.84)	0.31
Moderate to severe, 12 months	43/139 (31)	22/135 (16)	2.41 (1.24 to 4.70)	0.01
Neuropathic pain, DN4 positive:			Adjusted odds ratio (95% CI)	
6 weeks	21/150 (14)	24/153 (16)	0.73 (0.22 to 2.45)	0.61
6 months	29/133 (22)	26/145 (18)	1.64 (0.63 to 4.23)	0.31
12 months	32/139 (23)	22/135 (16)	1.29 (0.45 to 3.69)	0.64
Mean (SD) arm symptoms, FACT-B+4:			Adjusted mean difference (95% CI)	
6 weeks	4.5 (4.4)	4.1 (3.8)	−0.48 (−1.40 to 0.43)	0.30
6 months	4.7 (4.4)	3.4 (3.4)	−1.11 (−2.01 to −0.21)	0.02
12 months	5.4 (5.2)	3.4 (4.0)	−2.02 (−3.11 to −0.93)	<0.001
Lymphoedema, LBCQ:			Adjusted odds ratio (95% CI)	
6 weeks	20/150 (13)	22/153 (14)	1.07 (0.52 to 2.24)	0.85
6 months	32/133 (24)	29/145 (20)	0.82 (0.43 to 1.56)	0.55
12 months	36/139 (26)	33/135 (24)	1.17 (0.62 to 2.23)	0.62

DN4=Douleur Neuropathique-4 (positive neuropathic pain=score >3); FACT-B+4=Functional Assessment of Cancer Therapy-Breast+4 arm symptom scale; LBCQ=Lymphoedema and Breast Cancer Questionnaire (positive symptoms=arm both heavy and swollen).

* Numerical rating scale: acute and chronic postoperative pain. Moderate to severe pain=4-10.

We observed no differences in the rate of neuropathic pain, wound related complications (supplementary table S2), surgical site infection, lymphoedema, or other complications between treatment groups at any time point. No serious adverse events were reported. Physical health related quality of life scores were higher after exercise compared with usual care at both six months (adjusted mean difference in SF-12 physical component summary score 2.73, 0.24 to 5.21; P=0.03) and 12 months (4.39, 1.74 to 7.04; P<0.001) (table 4). We found no differences in mental health scores by treatment group over time. Women randomised to exercise were more confident in their ability to return to usual activities and regular physical activity compared with usual care participants, across all time points, although we observed no differences in self-reported activity of walking or strenuous activity between treatment groups (supplementary tables S3 and S4).

**Table 4:** Health related quality of life (SF-12) scores by treatment group

	Usual care	Exercise	Unadjusted estimate (95% CI)	P value	Adjusted estimate (95% CI)	P value
**6 months**	(n=133)	(n=145)				
Mean (SD) PCS; (missing)	43.2 (11.2); (5)	45.9 (9.5); (9)	2.73 (0.21, 5.25)	0.03	2.73 (0.24 to 5.21)	0.03
Mean (SD) MCS; (missing)	45.9 (11.1); (5)	48.0 (9.8); (9)	2.11 (−0.42, 4.64)	0.10	2.12 (−0.37 to 4.61)	0.09
**12 months**	(n=139)	(n=135)				
Mean (SD) PCS; (missing)	43.8 (11.5); (7)	48.1 (10.0); (10)	4.30 (1.63, 6.97)	0.002	4.39 (1.74 to 7.04)	<0.001
Mean (SD) MCS; (missing)	46.6 (11.2); (7)	48.7 (10.0); (10)	2.10 (−0.51, 4.71)	0.11	1.99 (−0.58 to 4.57)	0.13

Adjusted for age, baseline SF-12 score, breast surgery, axillary surgery, radiotherapy, and chemotherapy.

MCS=mental component summary score; PCS=physical component summary score.

**Fig 3:**
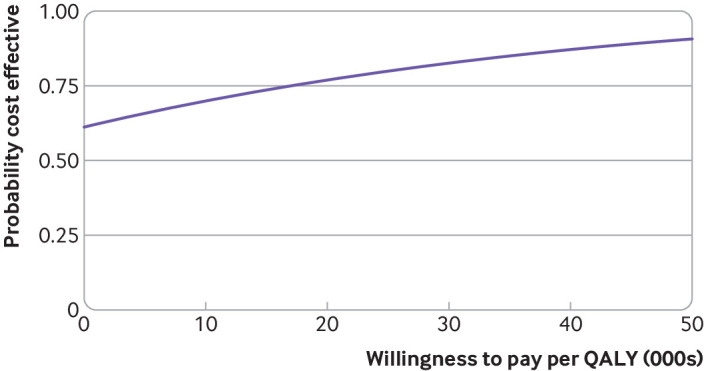
Cost effectiveness acceptability curve. Probability of cost effectiveness of exercise programme at alternative willingness to pay thresholds for an additional quality adjusted life year (QALY) held by decision makers. Increased values indicate higher probabilities of cost effectiveness for intervention programme

The exercise programme cost, on average, an additional £129 (€152; $178) per participant. When we considered all healthcare and personal social services costs, the incremental average cost was –£387 (95% confidence interval –£2491 to £1718) for the exercise group compared with the usual care group, representing a cost saving. When we controlled for baseline utility values, the exercise programme accrued an average 0.029 (95% confidence interval 0.001 to 0.056) more QALYs than usual care. This was a statistically significant increase (P=0.04). At the cost effectiveness threshold values of £20 000 and £30 000 per QALY specified by NICE, the probability was 78% and 84%, respectively, that exercise was the more cost effective of the two arms (fig 3). The probability of cost effectiveness at a willingness to pay threshold of £20 000 per QALY increased to 97% when we excluded the high cost cancer treatment (chemotherapy, radiotherapy), which had driven much of the uncertainty from the primary analysis. These findings remained robust to pre-specified sensitivity analyses (supplementary materials).

## Discussion

The structured PROSPER exercise programme improved upper limb function, postoperative pain, arm symptoms, and physical quality of life at 12 months, compared with usual care alone, in women at high risk of upper limb disability after breast cancer treatment. We found that our physiotherapy led exercise programme, introduced within seven to 10 days of breast cancer surgery, did not increase wound related complications, neuropathic pain, or lymphoedema symptoms at one year. Participants exceeded predefined clinically meaningful changes for upper limb function, and we observed lower rates of chronic postoperative pain and improved health related quality of life related to physical functioning after exercise than after usual care.

We included women undergoing contemporaneous cancer treatment. Axillary clearance procedures remain largely unchanged over recent decades, whereby tissues, including lymph nodes, bounded by the axillary vein, latissimus dorsi, chest wall, and pectoralis muscles, are removed. In addition to disturbance of the lymphatic system, putative mechanisms for postoperative morbidity include damage to the intercostobrachial nerve, increasing the risk of chronic neuropathic pain.^29^ Estimates for postoperative upper limb morbidity vary, although studies consistently report higher rates of shoulder impairment and chronic pain after axillary node clearance than after sentinel lymph node biopsy.^2^ ^30^ Radiation is independently correlated with upper limb morbidity: a network meta-analysis including 21 trials found that patients treated with axillary node clearance and radiotherapy targeting the supraclavicular area and chest wall were at increased risk of developing lymphoedema, with the greatest risk among those receiving regional node irradiation.^31^

### Comparison with other studies

Systematic reviews highlight the paucity of high quality evidence on the timing, safety, and optimal content of postoperative exercise after non-reconstructive breast cancer surgery.^4^ ^6^ A meta-analysis reported low quality evidence for the effectiveness of early rehabilitation on upper limb function up to six months postoperatively (three trials; total 526 participants, of whom 154 allocated to exercise).^7^ Low level evidence also exists for the safety of muscle strengthening after axillary node clearance (two trials; 422 participants; follow-up six months). We aimed to fill this gap by investigating the efficacy of early structured rehabilitation on functional and health related outcomes over one year. We measured upper limb function with the DASH, which has been shown to detect and differentiate changes in disability over time after surgery and for other upper extremity musculoskeletal disorders. The accepted minimally important clinical difference for people with painful disability is 10, but we accepted a smaller difference of seven as the study hypothesis was the prevention of post-treatment upper limb impairment. Most women in our study did not have severe upper limb dysfunction preoperatively, reflected in our sample’s mean baseline DASH scores (mean DASH 19), compared with studies of other conditions such as shoulder arthroplasty (mean DASH 64) or elbow arthroplasty (mean DASH 59).^24^ An improvement in DASH of five to 10 points indicates moderate improvement.^25^ Our intention to treat analyses suggests an observed effect size of 0.4, which may be considered modest but clinically worthwhile, given the pragmatic design and extended follow-up.^32^ Furthermore, our complier average causal effect analyses showed a 9 point change in DASH at 12 months among participants who were fully adherent to the exercise programme. Participants’ adherence was high, with 75% (143/191) attending the minimum three physiotherapy contacts. Strength exercises, using resistance bands, introduced at one month postoperatively were not associated with increased risk of lymphoedema, although we are aware that a risk of late onset lymphoedema, developing beyond 12 months, remains. We observed early benefits for some but not all secondary outcomes, with lower pain scores at six weeks and fewer arm symptoms at six weeks and six months in participants randomised to exercise (table 3). Fewer women allocated to exercise reported clinically meaningful moderate to severe intensity pain at one year (exercise 16% versus usual care 31%; table 3). We found no differences in rates of chronic neuropathic pain, concluding that early, progressive mobilisation was safe over the short and longer term. However, our exercise programme did not affect physical activity at one year: daily walking and strenuous activity levels were similar across the groups, with only one third of all women participating in strenuous sport on a weekly basis by one year (supplementary table S4). These activity levels are below international recommendations for physical activity.^33^

A systematic review found contrasting evidence for the cost effectiveness of exercise rehabilitation after breast cancer surgery, with only one Australian trial reporting health consequences expressed in QALYs over a 12 month horizon (sample size 194; mean QALY difference 0.009).^8^ Ours is the first UK cost effectiveness study, and we found that implementation of exercise was low cost (mean £129 per participant) and was associated with lower overall healthcare and personal social service costs and improved health related quality of life compared with usual care. Given that EQ-5D-5L scores were diverging in favour of exercise at 12 months, these are conservative estimates, and concluding that this would increase if the time horizon was extended is reasonable, assuming that the exercise programme follows the trajectory of continuing to accrue more QALYs than usual care. Much of the uncertainty surrounding cost effectiveness estimates was driven by the large and variable costs of other cancer treatments, including adjuvant chemotherapy, that might not be directly related to upper limb dysfunction. On removal of these costs, the probability that the intervention was cost effective at the £20 000 per QALY threshold was 97%.

### Strengths and limitations of study

The strengths of this study include the substantially larger sample size compared with previous trials, long follow-up period, high adherence to the intervention, multicentre involvement, and robust allocation concealment. The standardised exercise protocol was delivered by NHS physiotherapists from 17 different cancer units serving geographically diverse localities across England. The programme was co-developed with patients and clinical experts, resulting in a theoretically informed, fully manualised exercise intervention, incorporating behavioural change strategies and principles of exercise prescription based on the American College of Sports Medicine and American Cancer Society guidelines for cancer survivors.^10^ ^14^ Quality was assessed across all centres, so we are confident of treatment fidelity and avoidance of drift from the protocol by therapists.

Limitations of PROSPER include the lack of objective measurement of the secondary outcome lymphoedema by water displacement, arm circumference, or instrumental measurements, although this was not the primary purpose of our intervention. We used the validated patient reported Lymphoedema Breast Cancer Questionnaire,^21^ which has been shown to correlate with early onset of lymphoedema and is also being used as a primary outcome in other recently funded lymphoedema trials.^34^ Participants and physiotherapists were not masked to treatment, but this is an unavoidable limitation of therapy trials. We anticipated 25% loss to follow-up but observed slightly higher follow-up rates than predicted (78%); our participants were well matched with regards to cancer treatment, and losses were equally distributed by treatment allocation. Most drop-out occurred over the first six months, during active cancer treatments, with minimal loss thereafter. Imputation methods to assess the effect of data missingness did not alter the strength or direction of estimates of effect. Despite attrition, our trial was adequately powered for the primary outcomes, as the required sample size was 242 participants. Finally, our economic sensitivity analysis that used hospital episode statistics data was performed on a subset of participants and therefore may not be fully representative of the whole sample. However, these data capture only hospital admissions and therefore will have missed those costs (savings) most likely to be attributable to the exercise intervention.

### Implications for policy and practice

The PROSPER structured exercise programme introduced at one week postoperatively was safe to deliver, clinically impactful, and cost effective, providing the best quality evidence to date in support of prescription of early exercise for women at high risk of shoulder problems and upper limb morbidity after non-reconstructive breast cancer treatment. Future research directions could evaluate application of our preoperative screening criteria for the identification of women at higher risk of developing post-treatment limb related disability who could benefit from this cost effective exercise programme.

### Conclusions

We found robust evidence that early, structured, progressive exercise is safe and clinically effective for women at high risk of developing shoulder and upper limb problems after non-reconstructive breast surgery. The PROSPER exercise programme improved upper limb function at one year after breast cancer surgery and was cost effective compared with usual NHS care. Our manualised exercise intervention is suitable for wider implementation in clinical practice.

## What is already known on this topic

Upper limb disability is common after breast cancer treatment targeting the axilla, with up to one third of women experiencing problems postoperatively
Systematic reviews highlight the paucity of high quality trials, and uncertainty remains about whether early postoperative exercise may benefit patients at high risk of disability
No UK studies have assessed the clinical effectiveness or cost effectiveness of preventive strategies for patients at high risk of developing upper limb related disability after breast cancer treatment

## What this study adds

Early, structured exercise was safe, and women had better arm function and health related quality of life, with less pain and limb related disability over one year compared with usual care
The PROSPER exercise programme was clinically impactful and cost effective
This trial provides the best quality evidence to date in support of early exercise for women at high risk of shoulder problems after breast cancer treatment

## Data Availability

All data requests should be submitted to the corresponding author for consideration. Access to anonymised data may be granted following review. Data will be shared, with investigator support, after approval of a proposal, with a signed data access agreement. The study protocol and intervention materials are available online.

## Web extra

Extra material supplied by authors

Web appendix: Supplementary materials

## Notes

Contributors: JB, EW, SL, RL, AMT, and SP were responsible for securing funding, design of the trial and intervention, and oversight of study management. RL oversaw statistical analysis undertaken by PM and AH. AC and SP were responsible for health economic analysis. BM and JB oversaw delivery of the intervention. SR coordinated patient representation and qualitative research. EP coordinated trial management. All authors were responsible for interpretation of data and for approving the draft manuscript. JB is the guarantor. The corresponding author attests that all listed authors meet authorship criteria and that no others meeting the criteria have been omitted.

The lead author affirms that the manuscript is an honest, accurate, and transparent account of the study being reported; that no important aspects of the study have been omitted; and that any discrepancies from the study as planned (and, if relevant, registered) have been explained.

Dissemination to participants and related patient and public communities: We had invited trial participants to a dissemination event in March 2020, but this was cancelled owing to covid-19. We will publish a plain language summary of study results on the University of Warwick Clinical Trials Unit website for trial participants and distribute it to cancer units and physiotherapy departments involved in the research.

Provenance and peer review: Not commissioned; externally peer reviewed.
