# Supplementary material for: Exercise versus usual care after non-reconstructive breast cancer surgery (UK PROSPER): multicentre randomised controlled trial and economic evaluation
Source: BMJ. 2021 Nov 11;375:e066542. doi: 10.1136/bmj-2021-066542 (PMC8579424; doi:10.1136/bmj-2021-066542)
Supplement: Supplementary file 1 — Web appendix: Supplementary materials [file bruj066542.ww1.pdf]

Supplementary appendix – PROSPER (Bruce et al)

The PROSPER exercise intervention manual will be available from: https://wrap.warwick.ac.uk/cgi/users/home?screen=EPrint::View&eprintid=144049.

The PROSPER exercise training course will be available from: https://www.futurelearn.com/courses/prevention-of-shoulder-problems-prosper-programme/1

Box S1. Screening checklist for preoperative shoulder problems

Existing shoulder problems included any patient with a history of shoulder surgery, shoulder trauma injury (fracture or dislocation), frozen shoulder, osteoarthritis or rheumatoid arthritis affecting the shoulder, non-specific shoulder pain, stiffness, or weakness. Any patient with restricted range of shoulder movement or decreased shoulder function before surgery was eligible.

Questions to screen for functional problems:
Q. can the patient wash their hair without any problems?
Q. can the patient wash their back without any problems?
Q. can the patient reach up to place an object on a high shelf?
If yes, problems with one or more of the above, eligible for inclusion.
Also refer to surgical and radiotherapy criteria.[1]

Table S1. Disability of arm, shoulder and hand (DASH) subscales by treatment arm over time

	Usual care mean (SD)	Exercise mean (SD)	Unadjusted MD (95% CI)	P value	Adjusted* MD (95% CI)	P value
**DASH-AL**						
6 months	20.0 (20.8)	16.5 (17.8)	-3.47 (-8.25, 1.31)	0.15	-5.21 (-9.78, -0.63)	0.03
12 months	22.6 (23.3)	15.4 (18.2)	-7.23 (-12.30, -2.17)	0.005	-8.04 (-12.93, -3.14)	0.001
**DASH-PR**						
6 months	19.7 (21.4)	16.1 (17.2)	-3.60 (-8.37, 1.16)	0.14	-4.25 (-8.81, 0.31)	0.07
12 months	19.0 (22.4)	14.9 (20.2)	-6.08 (-11.28, -0.88)	0.02	-5.77 (-10.67, -0.88)	0.02
**DASH-I**						
6 months	23.2 (20.3)	20.9 (17.7)	-2.24 (-6.83, 2.36)	0.34	-2.94 (-7.77, 1.88)	0.23
12 months	26.8 (24.5)	19.1 (19.6)	-7.65 (-13.00, -2.33)	0.005	-7.15 (-13.19, -1.11)	0.02

*Adjusted for age, baseline DASH subscale score, breast surgery, axillary surgery, radiotherapy and chemotherapy. Higher scores indicate greater disability. AL= activity limitation; PR = participation restriction; I =impairment.

Table S2. Wound-related outcomes at six weeks

	Usual care N (%)	Exercise N (%)
Wound fully healed	122/150 (81.9)	126/153 (82.9)
Doctor-diagnosed SSI	40/150 (26.8)	38/153 (25.0)
Patient-reported SSI	40/150 (27.5)	39/153 (25.7)
Antibiotics prescribed for SSI	47/150 (31.8)	40/153 (26.8)
Any other complication	58/150 (39.5)	61/153 (41.2)
Wound seroma	31/150 (20.7)	33/153 (21.6)

Table S3. Confidence in ability to return to activities, by treatment arm over time

	Usual care	Exercise	Unadjusted MD (95% CI)	P-value	Adjusted MD (95%	P value
**6 weeks, N**	150	153				
Usual activities, mean (SD) [missing]	8.2 (2.4) [3]	8.8 (1.9) [0]	0.59 (0.10, 1.07)	0.02	0.32 (-0.16, 0.81)	0.19
Regular PA, mean (SD) [missing]	7.6 (2.6) [1]	8.5 (1.8) [1]	0.87 (0.34, 1.41)	0.002	0.67 (0.13, 1.20)	0.02
**6 months, N**	133	145				
Usual activities, mean (SD) [missing]	7.9 (2.2) [18]	8.6 (1.7) [16]	0.77 (0.27, 1.26)	0.002	0.68 (0.22, 1.14)	0.004
Regular PA, mean (SD) [missing]	7.7 (2.2) [18]	8.3 (2.0) [16]	0.62 (0.09, 1.14)	0.02	0.57 (0.07, 1.09)	0.03
**12 months, N**	139	135				
Usual activities, mean (SD) [missing]	7.6 (2.6) [1]	8.5 (1.8) [1]	0.87 (0.34, 1.41)	0.002	0.67 (0.13, 1.20)	0.02
Regular PA, mean (SD) [missing]	7.4 (2.7) [1]	8.3 (2.1) [1]	0.87 (0.30, 1.45)	0.003	0.73 (0.17, 1.30)	0.01

Adjusted for age, baseline confidence score, type of breast, type of axillary surgery, radiotherapy and chemotherapy. PA=physical activity. Confidence scale NRS= numerical rating scale 0-10.

Table S4. Walking and strenuous activity in previous week by treatment group

	Usual care N (%)	Exercise N (%)	P value
**Days walking, at 6 weeks**, N [missing]	150 [3]	153 [1]	
Never/Seldom, 1-2 days	28 (18.7)	20 (13.1)	0.17
Sometimes/Often, 3-7 days	119 (79.3)	132 (86.3)	
**Days walking, at 6 months**, N [missing]	133 [5]	145 [4]	
Never/Seldom, 1-2 days	27 (20.3)	20 (13.8)	0.14
Sometimes/Often, 3-7 days	101 (75.9)	121 (83.4)	
**Days walking, at 12 months**, N [missing]	139 [2]	135 [0]	
Never/Seldom, 1-2 days	24 (17.3)	20 (14.8)	0.55
Sometimes/Often, 3-4 days	113 (81.3)	115 (85.2)	
**Strenuous activity*, at 6 weeks**, N [missing]	150 [3]	153 [1]	
Never	126 (84.0)	130 (85.0)	0.96
Any	21 (14.0)	22 (14.4)	
**Strenuous activity, at 6 months**, N [missing]	133 [5]	145 [4]	

Never	96 (72.2)	110 (75.9)	0.56
Any	32 (24.1)	31 (21.4)	
**Strenuous activity, at 12 months**, N [missing]	139 [1]	135 [2]	0.74
Never	96 (69.1)	90 (66.7)	
Any	42 (30.2)	43 (31.9)	

Strenuous activity = strenuous sport or recreational activity in previous week.

Economic Evaluation Appendix

Overview
A within-trial economic evaluation was conducted to estimate the cost-effectiveness of the PROSPER exercise programme compared to usual care after breast cancer surgery. The primary health economic analysis took the form of a cost-utility analysis, expressed in terms of cost per quality adjusted-life year (QALYs) gained and incremental net monetary benefit. The analysis adopted the intention-to-treat principle. In line with NICE guidance[2], the analysis was based on an NHS and Personal Social Services (PSS) perspective. The price year adopted for the analysis was 2015 which was when the trial intervention materials were developed. The health economic analysis used a 12-month time horizon and consequently no discounting of costs or outcomes was required. Multiple imputation was used to address missing data. Hierarchical linear models were used to analyse the single cost and QALY endpoints, whilst a hierarchical net benefit regression framework was used to jointly examine costs and consequences. Uncertainty around cost-effectiveness was characterised using net-benefit plots and cost-effectiveness acceptability curves (CEACs), in addition to multiple sensitivity analyses.

Measurement of resource Use, costs and outcomes
Intervention costs were captured using a combination of methods including case-report forms (CRFs), an adapted client-service receipt inventory (CSRI) at six months and 12 months follow up, and intervention delivery data collected by physiotherapists and the trial team.
The costs within the analysis were divided into four components:

- Direct intervention costs
- Broader health care/PSS costs
- Wider costs
- Set up costs

The primary analysis adopted an NHS and PSS perspective and was concerned with the costs of delivering the intervention within an NHS setting. Set up costs and wider costs were considered within sensitivity analysis.

Direct intervention costs
All participants received usual care which involved a five minute contact with a specialist breast cancer nurse who provided usual care leaflets (BCC6[3] and BCC151[4]). In addition to leaflets, the intervention group received a physiotherapist-led exercise programme. Resource use was captured prospectively alongside the trial and we summarise the collection of resource use components in Table S6.

Table S6. Resource use - intervention costs

**Direct intervention costs - usual care**		
**Resource type**	**Resource use**	**Unit cost source**
BCC6 leaflet	1 per participant	Trial team
BCC151 leaflet	1 per participant	Trial team
Nurse time to explain information	5 minutes per participant	PSSRU
**Direct intervention costs - exercise intervention**		
Resource type	Resource use	Unit cost source
BCC6 leaflet	1 per participant	Trial team
BCC151 leaflet	1 per participant	Trial team
Nurse time to explain information	5 minutes per participant	PSSRU
Patient exercise planner	1 per participant	Trial team
Your Exercise manual	1 per participant	Trial team
Physiotherapist preparation time	Treatment log	PSSRU
Physiotherapist appointment (length)	Treatment log	PSSRU
Equipment	CSRI/Exercise log	NHS Supply chain
Contacts between appointments	Treatment log	PSSRU

Broader healthcare costs
Healthcare resource use as described in Table S7 was captured primarily through the CRF at six and 12 months. Data on healthcare use were collected for: inpatient care, outpatient care, community health care, medication, and equipment provided. Hospital Episode Statistics (HES) data were obtained for 242 patients who had reached 12 months from randomisation by the end of the 2017-2018 financial year, for use in secondary analysis. The resource use data collected within the CRFs were the primary source of cost data within the trial. Other wider

costs considered within secondary analyses included out of pocket costs, privately purchased equipment, and private health care costs. A further analysis included set up costs, which included resource use associated with training physiotherapists.

Table S7. Resource use: broader healthcare, wider and intervention set up resource use

**Broader healthcare resource use**	
Resource type	Unit cost source
Inpatient and day hospital care	NHS reference costs
Outpatient care	NHS reference costs
Community health care	NHS reference costs/PSSRU
Medication	NHS prescription cost analysis
Equipment	NHS supply chain
**Other wider resource use**	
Resource type	Unit cost source
Wider healthcare	Stated within CRF
Employment impacts	Income lost stated within CRF
Private health care	Stated within CRF
**Intervention set-up resource use**	
Resource type	Unit cost source
Trainers time – trained on site	PSSRU
Trainers time – centrally trained	PSSRU
Trainees attendance	PSSRU

Measurement of outcomes

In line with NICE guidelines[2], quality adjusted life years (QALYs) were the primary outcome for the economic evaluation.

Estimating QALYs

To calculate QALYs, it was necessary to obtain health state values for trial participants over multiple time points. We used the EQ-5D-5L, a five-dimension measure of HRQoL recommended by NICE.[2,5] There are multiple value-sets that allow the calculation of utility values associated with each and every state generated by the EQ-5D-5L measure.[6] At the time of writing, NICE preferred the use of the Van Hout et al. algorithm[7], hence this value set was used to calculate utility values. Health states were measured prospectively using the EQ-5D-5L at three time points: baseline, six and 12 months. Health state values as measured by the EQ-5D-5L were combined with time to calculate QALYs by calculating the area under the curve using the trapezium rule.[8] This method assumes that the health states reported at each time point were linearly interpolated. Participants who died during follow up were given an EQ-5D-5L score of zero at subsequent follow ups beyond the date of death.

Cost-effectiveness analysis methods

Missing data and multiple imputation

The cost-effectiveness analysis combines multiple cost components and multiple EQ-5D-5L scores across time points, multiple imputation (MI) was necessary to avoid the pitfalls associated with complete case analysis with substantial missing data. Missing data were assumed to be missing at random. To maximise the use of available data, MI was conducted at the component level e.g. for each healthcare cost variable and EQ-5D-5L at each time point. Costs and EQ-5D-5L scores were imputed jointly using chained equations and predictive mean matching; the imputation model included age, ethnicity, marital status, employment status and recruiting site as co-variates. For 15 participants lacking co-variate data, these were dropped from the MI analysis. Given missing data was approximately 30%-35% for each cost component, a total of 35 imputations were calculated to produce 35 complete data sets. MI procedures were conducted within Stata 16.[9]

Analyses of resource use, cost and QALYs

Resource use between trial arms was examined using standard statistical methods: descriptively and using t-tests for continuous variables and chi-squared tests for categorical variables, these are extensive and reported elsewhere. Regression models using the MI data were used to examine the impact of the intervention on the single cost and QALY end points. Multi-level linear models which account for the hierarchical data structure by including random effect parameters were used to estimate the single economic end points. Following recommendations, we adjusted for baseline difference between the two arms in the analysis of QALYs by including the EQ-5D-5L as a co-variate.[2]

Estimating cost-effectiveness
To examine cost-effectiveness, it was necessary to jointly assess the incremental costs and incremental effects. The net-benefit regression framework was chosen to assess cost-effectiveness as it has several strengths: i) it transforms the cost/QALY data from a ratio into a continuous variable allowing for easier manipulation whilst often normalising the data; ii) by combining costs and outcomes, it can seamlessly account for correlation between the two end points; iii) it allows easy control for baseline and co-variate imbalances[10]; iv) it can correct for clustering using a multi-level framework, v) it effectively deals with uncertainty around the decision makers willingness to pay (WTP) for the health outcome of interest; vi) it facilitates the generation of cost-effectiveness acceptability curves (CEACs) to present decision uncertainty; and vii) it is relatively straightforward to implement within Stata using MI data.

Characterising uncertainty
CEACs are a graphical representation of the probability that an intervention is cost-effective at different levels of WTP. NICE recommend that WTP thresholds of £20,000 and £30,000 per QALY are included within the CEAC when assessing uncertainty.[2] For a range of WTP thresholds, including those specified by NICE, CEACs were created to characterise uncertainty within cost-effectiveness estimates.

Sensitivity analyses
Several sensitivity analyses were conducted to examine the uncertainty surrounding trial results. These sensitivity analyses included:

i) Complete case analysis. This analysis considered only complete cases.
ii) Cost per DASH point. Should the intervention arm be associated with higher costs than the usual care arm, then the cost per DASH point were to be estimated.
iii) Costing from a societal perspective. In this sensitivity analysis, wider societal costs are included within the cost-effectiveness analysis. This includes: NHS health costs, private costs and over the counter (OTC) medication.
iv) Incorporating training within the evaluation. Sites were trained both centrally and at hospital sites, this analysis used a conservative approach whereby it is assumed each site was trained separately with up to two trial staff undertaking training for four hours at each hospital site.
v) Excluding high cost cancer healthcare usage. This analysis limited costs to intervention costs, community care costs, outpatient physiotherapy, outpatient pain clinics, outpatient complementary therapies/exercise facilities, and analgesics.
vi) Using HES cost data instead of CSRI data for hospital costs. This sensitivity analysis re-ran the primary analysis for the 242 participants with 12 months of complete data post-randomisation, prior to the HES cut-off date (31/3/2018) and used HES data for costing hospital costs instead of CSRI inpatient and outpatient data. As these hospital data are obtained centrally, we assumed these data were complete. Inpatient spells during the study and other hospital-based care costs were estimated by linking hospital episode data with Health Resource Groups, using the Reference Cost Grouper software[11], and then costed using NHS reference costs.[12]

Results
Analysis of cost
Intervention costs associated with the intervention were relatively small (

Table **S8**). The mean cost of physiotherapy appointments for those in the intervention arm was £103. Both trial arms received information leaflets alongside a 5-minute discharge appointment, however, these contributed very little to cost. For the intervention arm, there were other small costs, such as personalised exercise planner and manual, and manuals for the physiotherapist, and Therabands, these again were relatively small (£26). The total direct incremental cost associated with the intervention compared to the usual care arm was £129. Breast cancer related treatment formed most of the hospital costs (both inpatient and outpatient) for both arms, with non-cancer related costs being relatively minor. Medication costs were high and variable in both arms reflecting the oft high cost nature of cancer therapeutics. When comparing the incremental cost between the two arms (Table S9), the intervention was -£387 (95%CI: -2491, 1717) compared to the control arm representing a cost saving. As represented by the wide confidence intervals, there was substantial uncertainty surrounding this figure driven by the high costs related to breast cancer treatment.

Table S8: Cost components (complete cases)

	Control		Intervention	
**Cost (all visits)***	**Mean (SD)**	**95% CI**	**Mean (SD)**	**95% CI**
Inpatient Cost: BC-related	1707.20 (2748.50)	1201.71, 2212.68	1638.14 (2345.37)	1210.54, 2065.73
Inpatient Cost (non-BC related)	149.46 (433.81)	68.60, 230.31	198.44 (852.21)	38.14, 358.74
Outpatient Cost (BC-related)	3637.72 (2494.08)	3150.28, 4125.16	3617.43 (2606.48)	3115.46, 4119.41
Outpatient Cost (non-BC related)	239.24 (466.88)	151.01, 327.46	455.93 (1239.94)	221.61, 690.24
Community care	530.56 (584.26)	417.50, 643.63	460.12 (583.61)	347.73, 572.52
Medication	3211.05 (9508.71)	1527.71, 4894.90	2876.37 (10,278.99)	1092.68, 4660.06
NHS equipment	45.62 (80.64)	30.52, 60.72	48.78 (80.23)	33.90, 63.67
Other equipment	121.78 (601.79)	9.10, 234.46	92.89 (530.82)	-5.61, 191.38
Private Care	44.57 (241.60)	1.80, 87.34	28.36 (163.69)	1.01, 55.71
Other societal costs	147.44, 320.27	81.48, 213.40	261.73, 865.30	85.46, 438.00
Intervention cost: Physiotherapist time	n/a	n/a	102.56 (44.37)	95.88, 109.24
Intervention costs: Manuals/Equip	n/a	n/a	£26.48 (n/a)	n/a
Leaflets and 5-minute appointment for all participants	11.08	n/a	11.08	n/a

*Costs in GPB £ for price year 2015, see NIHR for full disaggregated resource use by time point.

Table S9: Analysis of cost

Total NHS Costs	MD	SE	t-value	p-value	95% CI
Intervention	-386.78	1073.48	-0.36	0.72	-2491.18, 1717.62

Analysis of QALYs

Health utility at each time point is reported in Table S10**Error! Reference source not found.**. At baseline, there was a very slight imbalance between the two arms with the usual care arm having a mean utility score of 0.666 compared to 0.683 in the intervention arm. The period from baseline to six months is associated a small decrease in health utility in both arms (control = 0.648, intervention = 0.673). Between six months and 12 months the utility scores diverge with the intervention arm increasing to 0.705; in contrast the usual care arm deteriorated to 0.633. Thus by 12 months the intervention arm reported improved utility scores compared to baseline, whilst the usual care arm reported worse scores. The utility scores which use the MI data tells a similar story (Figure S1). Imputed utility scores at all time-points are near identical to the complete case data, however uncertainty surrounding those estimates is reduced as reflected by the slightly narrower confidence intervals.

Table S10: Health utility profiles

	**Control**		**Intervention**	
**Health status – Complete Case[1]**	**mean**	**95% CI**	**mean**	**95% CI**
EQ-5D Baseline	0.666	0.633, 0.699	0.683	0.651, 0.719
EQ-5D 6 months	0.648	0.611, 0.685	0.673	0.643, 0.702
EQ-5D 12 months	0.633	0.597, 0.669	0.705	0.670, 0.741

Health status – Imputed Data	mean	95% CI	mean	95% CI
EQ-5D Baseline	0.665	0.630, 0.700	0.685	0.651, 0.719
EQ-5D 6 months	0.636	0.598, 0.674	0.673	0.644, 0.701
EQ-5D 12 months	0.626	0.592, 0.660	0.693	0.658, 0.728

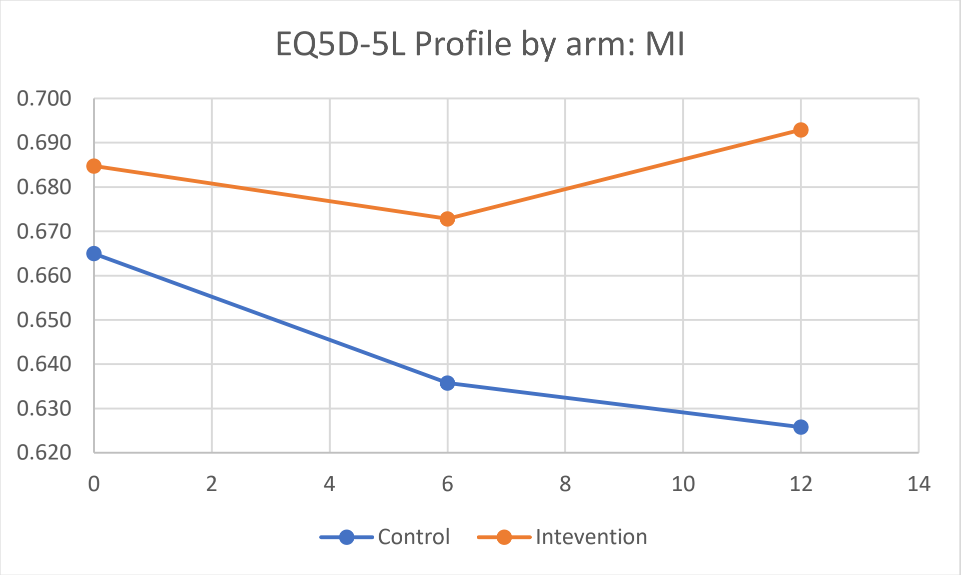

Figure S1: EQ-5D-5L Trajectory

The analysis of QALYs are shown in Table S11.. Using the MI data and controlling for baseline imbalance, the intervention arm accrued 0.029 (95% CI 0.001, 0.056) more QALYs than the usual care arm. This was a statistically significant increase (p=0.04).

Table S11. Analysis of QALYs

Incremental QALYs adjusted for baseline utility	MD	SE	t-value	p-value	95% CI
Intervention	0.029	0.014	2.050	0.041**	0.001, 0.056

Cost-effectiveness analysis

From the analysis of costs and analysis of QALYs it was evident that the intervention arm dominated the usual care arm. The joint cost-effectiveness results combining costs and QALYs within a net-benefit framework are shown in Figure S2 and Figure S3. As seen in Figure S2, net-benefit was positive at all levels of WTP including zero, this reflects the domination of intervention over the usual care arm. As represented by the lower 95% confidence interval for net-benefit being below zero, there is uncertainty surrounding the results. This aligns with the cost analysis that showed there was a large degree of uncertainty surrounding the incremental cost estimate.

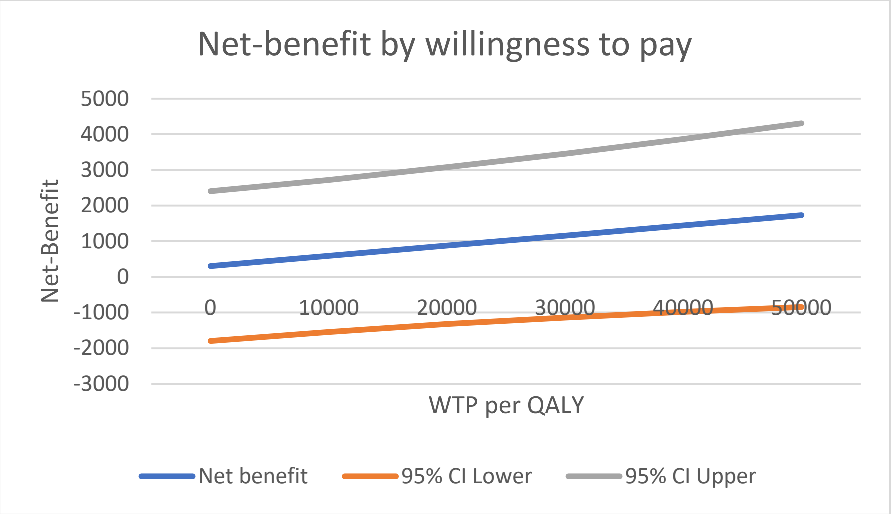

Figure S2: Net-benefit analysis

To examine the levels of uncertainty around the results, a CEAC was created (Figure S3). Even at a WTP of £0 there is still a 61% chance that the intervention is more cost-effective than the usual care arm. The CEAC is upward sloping due the positive co-efficient associated with incremental QALYs in the intervention arm. That is, as WTP for health benefits increase, so does the probability the intervention is cost-effective. At the NICE specified WTP threshold values of £20,000 per QALY and £30,000 per QALY there is a 78% and 84% probability that the intervention is the more cost-effective of the two arms. Given that EQ-5D-5L utility scores were diverging at the final time point it is reasonable to conclude that this probability would increase if the time horizon were extended beyond the trial as the intervention arm continues to accrue more QALYs than the usual care arm.

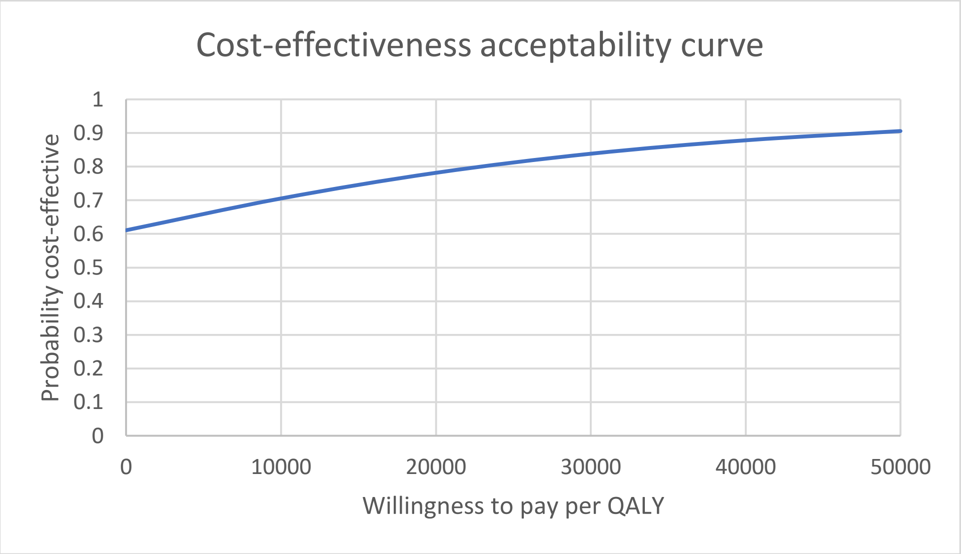

Figure S3: Cost-effectiveness acceptability curve

Sensitivity analyses

Summary results for the primary results and all sensitivity analyses are shown in Table S12. Sensitivity analysis one considers the cost-effectiveness results using the complete case data. The complete-case analysis provides supporting evidence for cost-effectiveness with there being a 65% chance the intervention is the more cost-effective option at a WTP of £20,000 per QALY rising to 68% at a WTP of £30,000 per QALY. Sensitivity analyses considered cost per DASH point. As reported in the main manuscript, the exercise intervention was associated with improved DASH score and lower costs. Given this, the intervention dominated the usual care

arm and so a cost per DASH point was deemed unnecessary due to the problems associated with interpreting a negative ICER. Secondary analysis 3 considers the impact of broadening the costing perspective from NHS and PSS to a societal perspective. This included other private costs healthcare costs, private equipment purchases, over the counter medication, and other costs. Income losses were omitted due to the lack of data for this variable. In terms of cost-effectiveness, this further strengthens the case for cost-effectiveness with the intervention continuing to dominate the usual care arm. In this analysis the intervention at a threshold of £20,000 per QALY has an 83% chance of being more cost-effective than the usual care arm when costed using this perspective. Sensitivity analysis 4 included training costs. Across the 17 sites, a total of 312 hours of training time were accounted for, this including the time of the trainers. The inclusion of these costs led to an increase in costs per intervention participant of £55.54. This had very little impact on the results of the cost-effectiveness analysis. In this analysis the probability of the intervention being cost-effective at a cost per QALY threshold of £20,000 falls marginally to 76.8%. Sensitivity analysis 5 considers a narrower costing perspective limited to those costs that are more plausibly to be impacted by shoulder problems, such as upper limb stiffness and pain, rather than cancer more generally. This led to much lower cost estimates with the mean costs falling to £732 (95%CI: £649, £815) per person. In this analysis the intervention arm is associated with an increased cost of £106 (95% CI: -£49, £262) with the probability of the intervention being cost-effective at a cost per QALY threshold of £20,000 per QALY increasing to 97%. This reflects the low costs and reduced uncertainty around cost-estimates within this analysis. This final sensitivity analysis used HES data to calculate hospital costs instead of CSRI data. Given the timescales involved for obtaining HES data within the trial timeline, it was only possible to obtain full 12-month data for 242 (63%) of the recruited participants. Within this analysis, costs were slightly higher within the intervention arm (+£166) with a great deal of uncertainty surrounding the estimate (95%CI: -£3849, £4181). This large increase in uncertainty is reflected in the CEAC with a 62% chance that the intervention is more cost-effective at a threshold of £30,000 per QALY.

Table S12. Cost-effectiveness summary

				Probability cost-effective at NICE threshold		Net Monetary Benefit at NICE threshold	
	**Incremental cost (95% CI)**	**Incremental QALYs (95% CI)**	**ICER £**	**£20,000/ QALY threshold**	**£30,000/ QALY threshold**	**£20,000/ QALY threshold**	**£30,000/ QALY threshold**
Primary analysis	-387 (-2491, 1718)	0.029 (0.001, 0.056)	Intervention dominates	78%	84%	874	1160
SA1: Complete case	-259 (-3609, 3092)	0.030 (0.002, 0.059)	Intervention dominates	65%	68%	676	888
SA2: Incremental cost per DASH point	-387 (-2491, 1718)	N/A	Intervention Dominates	N/A	N/A	N/A	N/A
SA3: Including societal costs	-642 (-2826, 1542)	0.029 (0.001, 0.056)	Intervention Dominates	83%	87%	1096	1378
SA4: Including training costs	-331 (-2436, 1773)	0.029 (0.001, 0.056)	Intervention dominates	77%	83%	819	1104
SA5: Attributable costs	111 (-44, 267)	0.029 (0.001, 0.056)	£3827 per QALY	97%	98%	524	847
SA6: HES	171	0.028	£6107	57%	62%	384	664

sub-sample (n=242)	(-3844, 4186)	(-0.006, 0.061)	per QALY				

Discussion

This economic evaluation examined the costs and outcomes associated with the PROSPER exercise intervention in comparison to usual care. A multi-level net-benefit regression framework was used to assess the cost-effectiveness of the intervention and to estimate the uncertainty surrounding the results. The results found that the exercise intervention was cost-effective compared to usual care, with the exercise intervention within the primary analysis having a 78% chance of being the more cost-effective option at the NICE cost-effectiveness threshold of £20,000 per QALY. The results were robust to a range of sensitivity analyses. Given that EQ-5D-5L utility scores were diverging at the final time point it is reasonable to assume that these estimates are conservative. This is reinforced by secondary analysis 5, which found there was a 97% chance of cost-effectiveness when excluding likely non-attributable costs, e.g. high cost cancer treatments and inpatient surgery, which drove much of the uncertainty around the cost estimates in the other analyses.

There were several limitations to this economic analysis. Whilst missing EQ-5D-5L data were relatively low, there was significant missing data for health care usage data as is common within trials. To address this, multiple imputation was used to make the most of available data whilst retaining uncertainty; this requires an assumption that data is missing at random which may not be the case. Although the cost-effectiveness estimates were favourable, there was large uncertainty surrounding incremental cost estimates. This was due to the high cost and variable nature of breast cancer treatment whereby certain cancer treatments unrelated to the rehabilitation of the shoulder post-surgery account for most of the costs. Consequently, we included a sensitivity analysis that included only those costs that might plausibly be related to shoulder pain and discomfort. In this analysis, there was much less uncertainty around cost estimates which resulted in a very high probability of the intervention being cost-effective (97%). The sensitivity analysis that used HES data was only performed on a subset of the data and therefore is not fully representative of the sample. Furthermore, it only captured hospitalisations and therefore will have missed those costs (savings) most likely to be attributable to the intervention.

This was a trial-based analysis, therefore a limitation to this is that the EQ-5D-5L utility scores had not converged by the final time point. Given that the exercise intervention arm were still in a better health state at the final time point and costs were largely upfront, it is likely that the strength of evidence for cost-effectiveness would be stronger still if longer term follow up was conducted. Linear interpolation was specified as the method for calculating QALYs. This is a limitation as the time between each follow-up was significant and trajectories may not follow a linear pattern. Given the prolonged nature of treatment in this cohort we however felt this was the best approximation with the data we had. Finally, there is still debate about the validity of the EQ-5D-5L. At the inception of the study this measure was recommended by NICE and hence was chosen to 'future proof' results. The use of the 3-level version however may have given slightly different results. Given the difference in QALYs between the two groups, we do not anticipate that this would have meaningfully changed the results.

References

1. Bruce J, Williamson E, Lait C, et al. Randomised controlled trial of exercise to prevent shoulder problems in women undergoing breast cancer treatment: study protocol for the prevention of shoulder problems trial (UK PROSPER). *BMJ Open* 2018; **8**(3): e019078.
2. National Institute for Health and Excellence (NICE). Guide to the Methods of Technology Appraisal. London, 2013.
3. Breast Cancer Care. Exercises after breast cancer surgery London: Breast Cancer Care 2013.
4. Breast Cancer Care. Your operation and recovery London: Breast Cancer Care 2013.
5. Herdman M, Gudex C, Lloyd A, et al. Development and preliminary testing of the new five-level version of EQ-5D (EQ-5D-5L). *Quality of Life Research* 2011; **20**(10): 1727-36.
6. Devlin N, Shah K, Feng Y, Mulhern B, van Hout B. Valuing health-related quality of life: an EQ-5D-5L value set for England. 2016.
7. van Hout B, Janssen MF, Feng YS, et al. Interim scoring for the EQ-5D-5L: mapping the EQ-5D-5L to EQ-5D-3L value sets. *Value in health: the Journal of the International Society for Pharmacoeconomics and Outcomes Research* 2012; **15**(5): 708-15.

8. Manca A, Hawkins N, Sculpher MJ. Estimating mean QALYs in trial-based cost-effectiveness analysis: the importance of controlling for baseline utility. *Health Econ* 2005; **14**(5): 487-96.
9. Stata statistical software: Release 16. 16 ed. College Station, TX. : StataCorp; 2019.
10. Hoch JS, Hay A, Isaranuwatchai W, et al. Advantages of the net benefit regression framework for trial-based economic evaluations of cancer treatments: an example from the Canadian Cancer Trials Group CO.17 trial. *BMC cancer* 2019; **19**(1): 552.
11. NHS Digital. Costing - HRG4+ 2016/17 Reference Costs Grouper. 2020. https://digital.nhs.uk/services/national-casemix-office/downloads-groupers-and-tools/grouper-and-tools-archive/costing-hrg4-2016-17-reference-costs-grouper (accessed 21/02/2020 2020).
12. (DH) DoH. NHS reference costs 2014 to 2015. London: Department of Health (DH); 2015.
